# Expression of the bitter receptor T2R38 in pancreatic cancer: localization in lipid droplets and activation by a bacteria-derived quorum-sensing molecule

**DOI:** 10.18632/oncotarget.7206

**Published:** 2016-02-05

**Authors:** Matthias M. Gaida, Christine Mayer, Ulrike Dapunt, Sabine Stegmaier, Peter Schirmacher, Guido H. Wabnitz, G. Maria Hänsch

**Affiliations:** ^1^ Institute of Pathology, University Hospital Heidelberg, Heidelberg, Germany; ^2^ Department for Gynecology and Obstetrics, University Hospital Heidelberg, Heidelberg, Germany; ^3^ Department for Orthopedics, University Hospital Heidelberg, Heidelberg, Germany; ^4^ Institute of Immunology, University Heidelberg, Heidelberg, Germany

**Keywords:** pancreatic cancer, bitter receptor, microbiome, ABCB1

## Abstract

T2R38 belongs to the family of bitter receptors and was initially detected in cells of the oral cavity. We now describe expression of T2R38 in tumor cells in patients with pancreatic cancer and in tumor-derived cell lines. T2R38 is localized predominantly intracellular in association with lipid droplets, particularly with the lipid droplet membrane. The receptor can be activated by the *bona fide* ligand for T2R38, phenylthiourea (PTU), and by N-acetyl-dodecanoyl homoserine (AHL-12), a quorum sensing molecule of *Pseudomonas aeruginosa*, the latter is the only known natural ligand for T2R38. In response to PTU or AHL-12, key transcription factors are activated including phosphorylation of the MAP kinases p38 and ERK1/2, and upregulation of NFATc1. Moreover, we found increased expression of the multi-drug resistance protein 1 (also known as ABCB1), a transmembrane transporter molecule, participating in shuttling of a plethora of drugs, such as chemotherapeutics or antibiotics. In conclusion, our data indicate a new, additional function of the taste receptor T2R38 beyond sensing ‘bitter’. Moreover, because T2R38 can be stimulated by a bacteria-derived signaling molecule the receptor could link microbiota and cancer.

## INTRODUCTION

Pancreatic ductal adenocarcinoma (PDAC; pancreatic cancer) is a highly aggressive tumor with an overall 5-years survival rate of approximately 5% [1]. Therapeutic options are rather limited, due to aggressive and invasive growth, early metastasis, and resistance to radiation and chemotherapy [2]. A characteristic feature of PDAC is the extensive desmoplastic stroma and the pro-inflammatory tumor microenvironment [3]. The link between this intratumor inflammation and cancer progression is not yet understood. Alterations of the cytokine profile of infiltrating immune cells are discussed [4], and more recently also the role of the intestinal microbiota [5, 6]. Although microbiota and host co-exist in a more or less symbiotic relationship, alterations within the microbiome or changes of life-style might off-set this balance, leading to a local immune response, inflammation, or metabolic changes, which in turn might promote cancer development [5, 6]. Interactions between tumor cells and bacteria-derived substances are not well understood, particularly in tissues lacking a microbiome, such as the pancreas. In that context, we were interested in receptors on tumor cells that recognize soluble, tissue-permeating bacteria-derived molecules. For the latter, we chose the quorum sensing molecule N-acetyl-dodecanoyl-homoserine lactone (AHL-12), which is produced and released by Gram-negative bacteria as an interbacterial signaling molecule, crucial for developing virulence factors. AHL-12, however, signals also to a wide variety of mammalian cells, including myeloid cells and epithelial cells [7, 8]. Due to its lipophilic nature, AHL-12 diffuses freely through membranes; more recently, however, T2R38 was identified as receptor for AHL-12 on epithelial cells and myeloid cells, linking T2R38 to the local host response to bacterial infection [7-11].

T2R38 belongs to the family of bitter receptors, originally identified on cells of the taste buds (reviewed in [12-14]). In humans, the family comprises approximately 25 bitter receptors, encoded from 43 genes. Some of the receptors can bind different structurally unrelated compounds, for others the natural ligands have not yet been identified [14, 15]. Of note, bitter receptors are also expressed by cells that are not part of the gustatory system, e.g. by airway epithelial cells, in the brain, ductal cells of the pancreas, or enteroendocrine cells in the colon and by myeloid cells [7, 8, 16-18]. Obviously, these receptors do not participate in taste perception, and their responsiveness to a wide variety of structurally unrelated substances, including amino acids, peptides or sugars, imply other, possibly regulatory, functions.

We now assessed expression of T2R38 in PDAC tissue and on a variety of tumor cell lines and assessed its biological function by use of AHL-12 as a natural ligand.

## RESULTS

### Expression of T2R38 in biopsies

**Figure 1:**
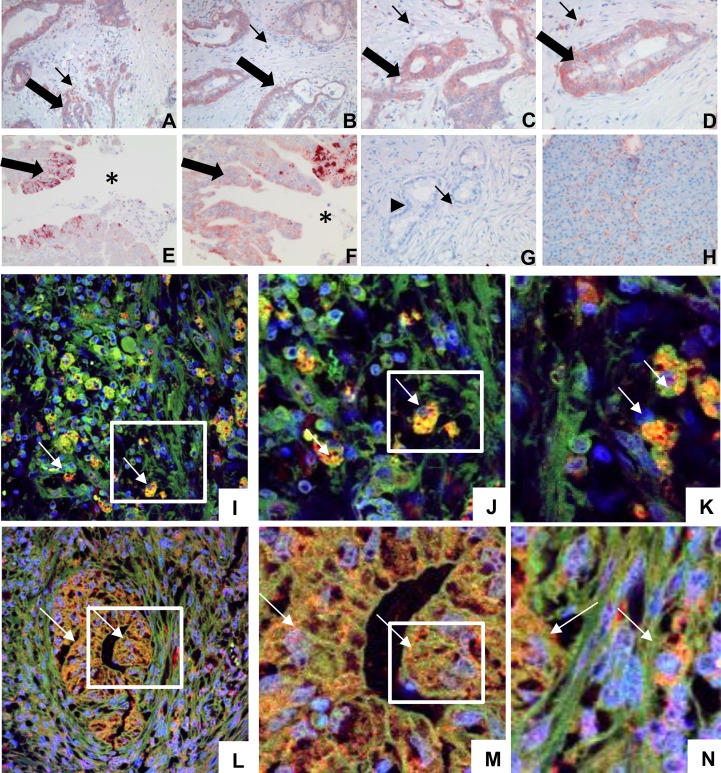
Expression of T2R38 in pancreatic tumor tissue and association with lipid droplets **A.**-**D.** Tissue biopsies of PDAC are shown, stained for T2R38 (two different tumors; magnifications: A,B: 100x; C,D: 200x) PDAC cells were positive for T2R38 (thick arrows), as were infiltrating immune cells (thin arrows). **E.**, **F.** T2R38 positive tumor cells (thick arrows) at the invasive front of the tumor in the duodenal wall (magnifications E: 200x; F: 400X). The gut lumen is marked by an asterisk. **G.** For comparison, a PDAC biopsy negative for T2R38 is shown (still with scattered T2R38 positive infiltrating immune cells marked by thin arrow; tumor marked by arrowhead; 200x), and in **H**. normal acinar pancreactic tissue, which is negative for T2R38 (200x). Panel **I.** shows infiltrating immune cells expressing T2R38 (green; the nucleus is stained in blue) in association with perilipin-2 (red) yielding yellow dots (digital zoom of two different selected areas are shown on **J.** and **K.** the arrows show examples of immune cells). **L.** A gland of a pancreatic ductal adenocarcinoma is shown; again expressing T2R38 (green) in association with perilipin-2 (red). **M.**, **N.** digital zoom images of two different selected areas. (The arrows show examples of tumor cells).

Expression of T2R38 and association with lipid droplets was examined in tissue derived from biopsies of patients with pancreatic ductal adenocarcinoma. In 69 of the 88 pancreatic cancer biopsies (78.4%) T2R38 was seen in tumor cells. The T2R38 signal was mainly located in the cytoplasm, and only rarely on the membrane. Staining intensity of the tumor cells was moderate, whereas the tumor infiltrating macrophages and neutrophils stained more prominently. T2R38 could also be detected at the invasive front of the tumor infiltrating the duodenal wall at the interface to the duodenal lumen. The surrounding normal tissue, mostly acinar cells, did not express T2R38 (examples in Figure 1A-1H). Staining intensity or frequency of T2R38 positive tumor cells did not correlate with clinical or pathological parameters or survival, nor did the number of infiltrating leukocytes. In tumor cells and in leukocytes T2R38 was co-localized with perilipin-2, which is an established marker for lipid droplets (Figure 1I-1N).

### Expression of T2R38 on tumors and tumor cell lines

**Figure 2:**
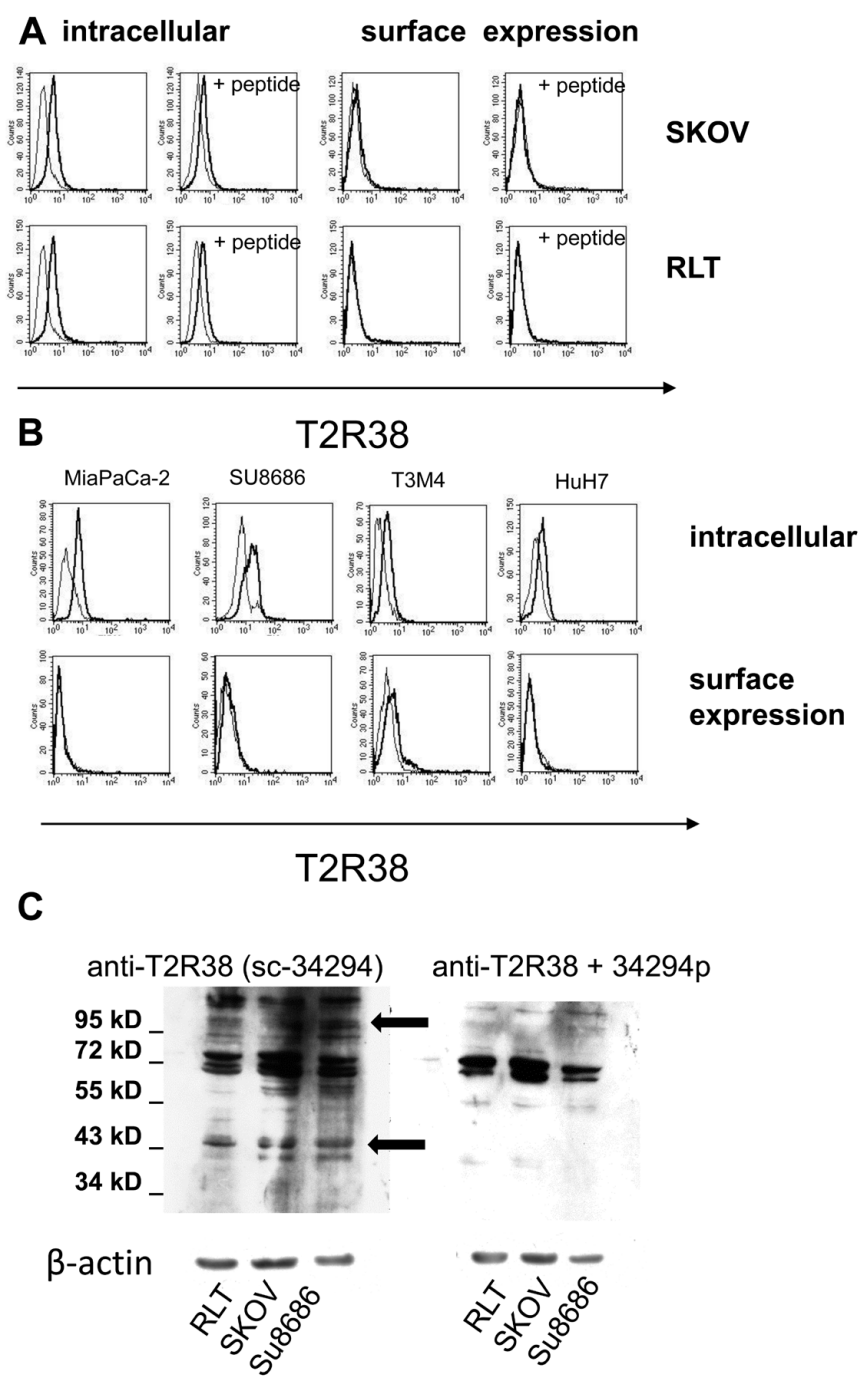
Expression of T2R38 on tumor cell lines **A.** By cytofluorometry intracellular expression and surface expression of T2R38 was tested on the cells lines indicated (left panels; the thick line shows antibody binding, the thin line the isotype control). Right panels: To verify specificity of the antibody binding (thick line), the cells were preincubated with the corresponding antigen peptide (thin line). **B.** Examples of the pancreatic cancer cells MiaPaCa-2, Su8686, T3M4 and the liver cancer cell line HuH7 are shown. **C.** By Western blotting expression of T2R38 was tested in whole cells lysates of RLT, SKOV, and SU8686. The antibody sc-34294 reacted with multiple proteins; inhibition by the antigenic peptide (34294p) revealed specific bands with an apparent molecular weight of 100 kDa and 38 kDa, respectively (marked by arrows).

By cytofluorometry, expression of T2R38 was tested on pancreas-derived cancer cells (SU8686, T3M4, MiaPaCa-2), and for comparison on RLT (a pancreatic stellate cell line), HuH7 (liver cancer cell line), and SKOV-3 (ovarian cancer cell) (Figure 2A, 2B). In all, T2R38 was present intracellular, whereas surface expression was weak (HuH7, SU8686) or absent. Specificity of the antibody reaction was confirmed by use of the antigenic peptide, which greatly reduced the antibody binding (on average: 57.7%; range: from 31.3% to 81.4%). Western blot revealed numerous protein bands; two of which could be inhibited by the antigenic peptide, indicating specific reaction with a protein at 38 kDa protein, which corresponds to the expected size. In addition, a 100 kDa form of T2R38 was found (Figure 2C). The latter is in line with published data, and is thought to represent a post-translationally modified form [7, 19].

### Association of T2R38 with lipid droplets

**Figure 3:**
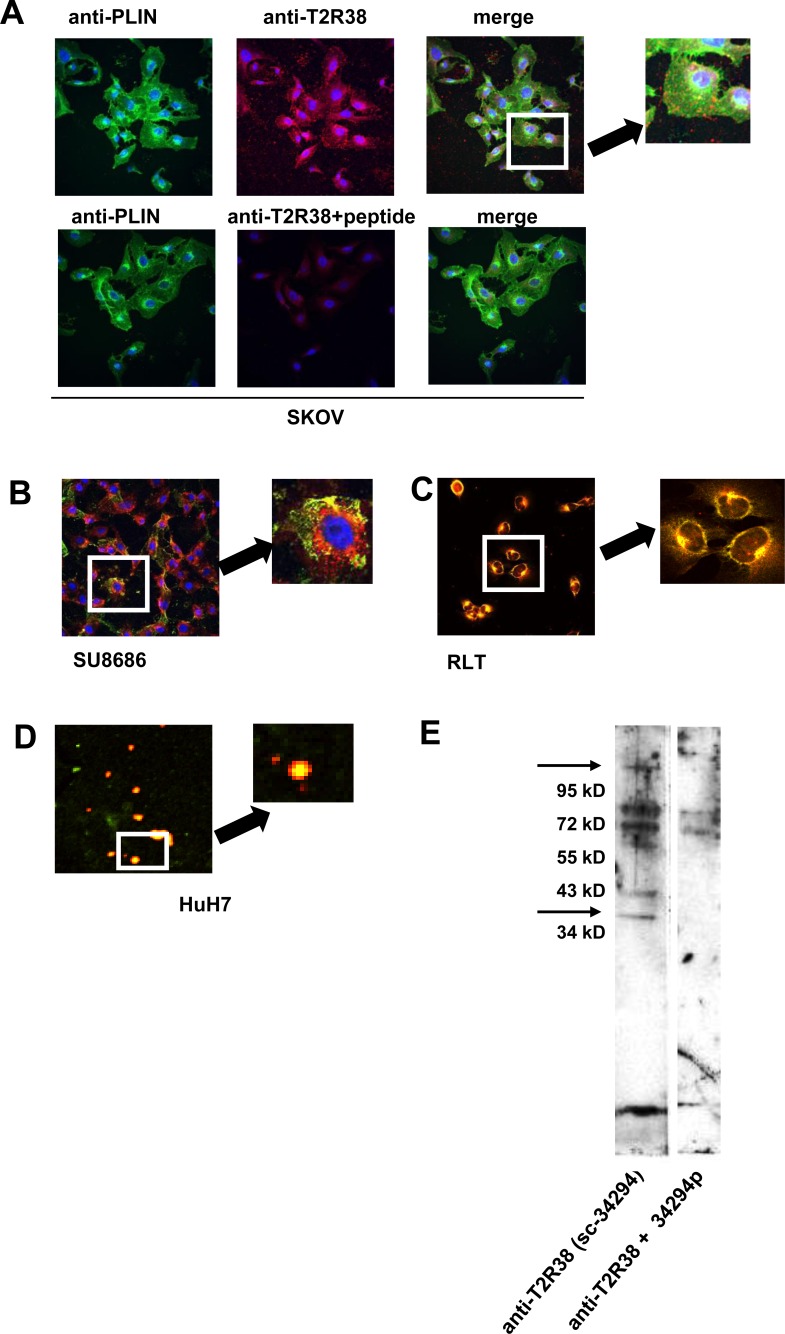
Association of T2R38 with lipid droplets **A.** upper panel: In SKOV, T2R38 (red) and perilipin-2 (PLIN; green) as marker for droplets were visualized using the respective antibodies; nuclei are stained in blue. On the right, the merged image indicates a co-localization of T2R38 and perilipin-2 (yellow dots). The lower panel shows the same experiments with cell preincubated with the antigenic peptide, which inhibited - as expected - binding of the antibody. **B.** shows the merged image for SU8686 (staining as above). **C.** In this experiment, the droplets were stained with Nile red, and again co-localization with T2R38 (green) was seen in the merged images (RLT cells). **D.** Droplets were isolated from cells, and stained with Nile red. T2R38 was visualized using anti-T2R38 (green). The merged images show co-lozalisation. **E.** By Western blotting, T2R38 was detected in the isolated droplets. Again, the antigen peptide inhibited the reaction of the antibody.

Analyses of cell lines by laser scan microscopy showed an association of T2R38 with the cell membrane, and throughout the cytoplasm in a more or less speckled pattern. As in the biopsies, T2R38 was found in lipid droplets, which were visualized either by staining with Nile red or by an antibody to perilipin-2, the latter a marker for lipid droplets [20] (Figure 3A, 3B, 3C). The association of T2R38 with lipid droplets was confirmed by isolating droplets from cell lines SU8686 and HuH7, and examining them for expression of T2R38 by laser scan microscopy and Western blotting (data for HuH7 are shown in Figure 3D, 3E).

### Activation of T2R38 by AHL-12 or PTU

**Figure 4:**
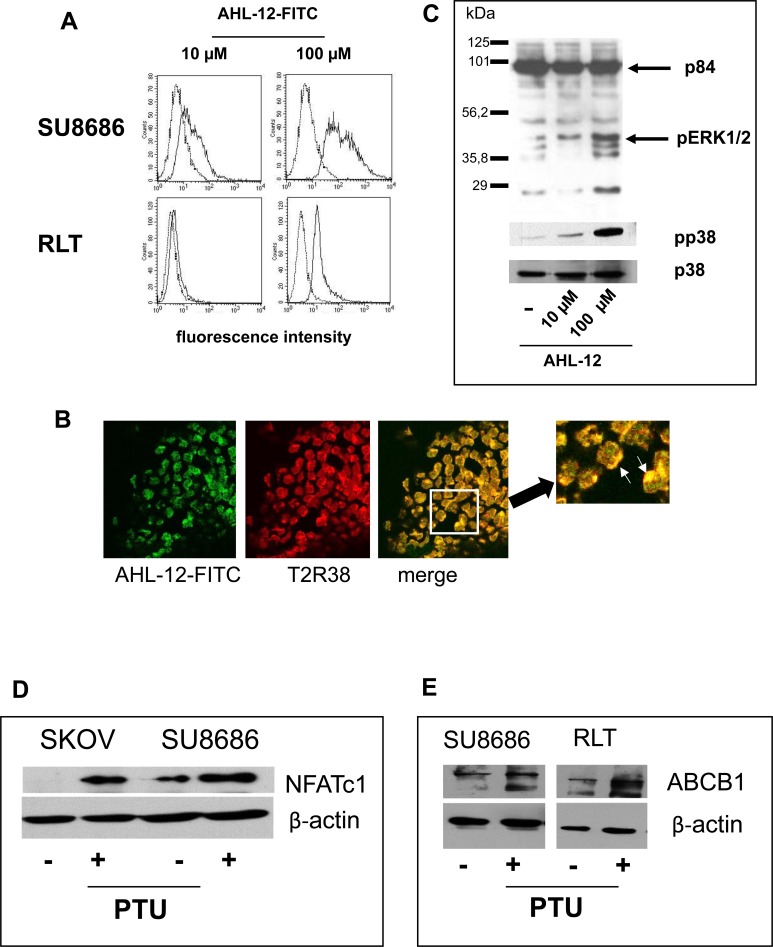
Binding of AHL-12 to cells and activation **A.** Cells were incubated with FITC-labelled AHL-12 for 30 min. at 4°C; the fluorescence associated with the cells was determined. In each panel, auto fluorescence of the cells is shown (left peaks in the histogram), and green-fluorescence (the right peak) indicating AHL-12-FITC binding. **B.** in a parallel experiments, cells were incubated with AHL-12-FITC and anti-T2R38 (red) and viewed by laser scan microscopy. Co-localisation of AHL-12-FITC with T2R38 was seen (marked on the digital zoom). **C.** By Western blotting phosphorylation of p38 and pERK (1/2) was seen following stimulation of cells with AHL-12 (shown is p38 and its phosphorylated form pp38; p84 was used as loading control), as was up-regulation of NFATc1 (SKOV: 23.9x; SU8686: 2.8x) **D.**, and of ABCB1 (SU8686: 1.8x; RLT: 2.0x) **E.** (β-actin was used as loading control).

Previous data by us and others identified the quorum sensing molecule of *P. aeruginosa* AHL-12 as a natural ligand for T2R38 [7, 8]. AHL-12 is known to bind to numerous target cells, such as neutrophils or macrophages [21, 22], and within 30 min to pancreatic tumor cells or pancreatic stellate cells, as seen by cytofluorometry (Figure 4A) or laser scan microscopy (Figure 4B). In consequence, the MAP kinases p38 and ERK1/2 are phosphorylated, and NFATc1 was upregulated (Figure 4C, 4D). In parallel to AHL-12, phenylthiourea (PTU) was used, as is a widely used specific though artificial ligand for T2R38. Again, p38 and ERK1/2 were phosphorylated, and NFATc1 expression was enhanced. Moreover, triggering T2R38 induced up-regulation of the multidrug-resistance protein ABCB1. (Figure 4E).

## DISCUSSION

Bitter receptors were initially detected in cells of taste buds of the oral cavity, as chemosensors for bitter tasting substances. In recent years, a broader distribution of some bitter receptors was reported, for example in neutrophils, breast cancer cell lines, enteroendocrine cells of the colon, or airway epithelial cells [7, 8, 16, 18, 23-25]. We now detected T2R38 in biopsies derived from patients with pancreatic cancer. Tumor cells expressed T2R38, but also the infiltrated leukocytes in the tumor microenvironment, whereas the surrounding normal pancreatic acinar cells were negative. Neither staining intensity nor the frequency of T2R38 positive tumor cells correlated with clinical or pathological parameters, or with survival, presumably due to the fact that especially pancreatic tumors are rather heterogeneous in their composition.

T2R38 was mainly located in the cytoplasm in close association with lipid droplets. Lipid droplets (also known as lipid bodies or liposomes) were originally described as storage compartments for lipids. More recently, they are recognized as functional cell organelles, equipped with specific proteins, and participating in lipid turnover, and in the generation of inflammatory lipid mediators, such as leukotrienes (reviewed in [13, 26-29]). Additional functional activities are to be expected, because lipid droplets also contain molecules that are not per se involved in lipid metabolism, for example cytokines or metabolites involved in intracellular trafficking [30]. Possibly, lipid droplets are especially suitable for proteins that are predestined for binding in a lipophilic environment ([30], reviewed in [13, 26, 28]), such as T2R38.

T2R38 on pancreatic cells is functional. The bitter tasting chemical PTU that is widely used as specific ligand for T2R38 and the bacterial quorum-sensing molecule AHL-12, which is so far the only known natural ligand for T2R38, activated the MAP kinases p38 and ERK1/2, and up-regulated NFATc1. This signaling pathway concurs with G-protein-dependent signaling, and with the activation pathway that has been described for AHL-12 [21, 22, 31]. Although G-protein-dependent signaling implies a surface receptor, as do the pathways activated by AHL-12 [21, 22, 31], we found T2R38 predominantly intracellular. Possibly, T2R38 can shuttle between the storage site and the cell membrane. In principle, T2R38 can be expressed on the cell surface, for example on myeloid cells, and also our uptake studies with labeled AHL-12 show its co-localization with T2R38 on the surface. On the other hand, because AHL-12 is lipophilic, it is possible that it diffuses into the cell, and binds intracellular.

The biological role of T2R38 beyond tasting “bitter” is still under investigation. A role in local host defense has been proposed, based on data showing expression of T2R38 on airway epithelial cells and on phagocytic cells, and up-regulation of defense-relevant functions by AHL-12 [7, 9, 32-35]. Moreover, receptor allotypes with low binding AHL-12 capacity predispose to infection [8, 11], in line with data shown for other taste receptors [36, 37].

The role of T2R38 in tumors is less apparent. *Clark* et al. suggested that bitter-tasting substances may have specific physiological effects on cells carrying bitter receptors, possibly underlying the “off-target drug effects” of bitter-tasting pharmaceuticals [38]. In line with this hypothesis, influence on tumor cell biology by triggering T2R38 is feasible, for example the response of tumor cells to chemotherapeutics might be decreased, because the multi-drug resistance protein 1 (ABCB1) was up-regulated in response to the established ligands of T2R38, PTU or AHL-12. ABCB1 is a transmembrane transporter that functions as an efflux pump for potentially toxic substances, including chemotherapeutics (reviewed in [39, 40])

Whether bitter receptor ligands come into contact with tumor cells in pancreatic cancer in sufficient concentrations is debatable. AHL-12 and other molecules derived from the gut microbiome might be distributed *via* the portal venous blood supply. Moreover, in advanced tumor stages, extensive tissue damage and loss of epithelial barriers occurs, and bacterial infections are a common when a pancreatic cancer infiltrates the gut. On the other hand, AHL-12 may not be the only ligand for T2R38, because promiscuity - even for structurally unrelated substances - has been described for a number of bitter receptors [15], and therefore it is feasible that T2R38 responds to bitter tasting substances, for example, of food. Data showing that polymorphisms of T2R38 (responder versus non-responder allotypes with regard to the artificial ligand PTU) are linked to the occurrence of colon cancer [41] support a role of food ingredients.

A link between bitter tasting substances and tumor biology has been suggested before. For example, increased coffee consumption reduced the risk of liver cancer [42], and lupulones from hops or epigallocathechin gallate from green tea induce apoptosis of tumor cell lines [43, 44]. In line with tumor control *via* bitter receptors are data showing that normal breast epithelial cells express T2R38, whereas tumor cell lines downregulate the receptor [45].

In summary, our data demonstrate expression of the bitter receptor T2R38 in human pancreatic cancer. The biological relevance of the extragustatory expression of T2R38 is still under investigation; effects of gut microbiome derived mediators are feasible, considering that T2R38 responds to a bacteria-derived molecule. Engagement of T2R38 may take part in in pancreatic cancer progression *via* the induction of chemoresistance mechanisms.

## MATERIALS AND METHODS

### Cell lines

RLT cell line was a gift of Dr. Ralf Jesenofsky, University Hospital of Mannheim, Medical Faculty Heidelberg, Germany). The cells were propagated in DMEM High Glucose (Cell Concepts, Umkirch, Germany), containing foetal calf serum (10 %), L-glutamine (1 %). HuH7 cell line (JCRB Cell Bank, Osaka, Japan) were propagated in DMEM High Glucose (Cell Concepts, containing foetal calf serum (10 %), L-glutamine (1 %) and penicillin-streptomycin (1 %). SU8686, MiaPaCa-2 (ATCC, Rockville, USA), and T3M4 (a gift of European Pancreas Center, Heidelberg, Germany) were propagated in RPMI, containing foetal calf serum (10 %), L-glutamine (1 %) and penicillin-streptomycin (1 %). SKOV-3 (ATCC) were propagated in McCoys5A (Biochrom, Berlin, Germany), containing fetal calf serum (10 %), L-glutamine (1 %) and penicillin-streptomycin (1 %)

### Cytofluorometry

To determine the expression of surface receptors, cells (10^6^/ml) were incubated with anti-T2R38 (sc-34294, Santa Cruz, Heidelberg, Germany (2-6 μg), and PE-labelled anti-goat-IgG (PA129953, Thermo Scientific, Waltham, MA, USA) as secondary antibody. The specificity of the antibody was verified using the peptide sc-34294p (2 μg). Cells were subjected to cytofluorometry using FACSCalibur and CellQuest Pro software (Becton Dickinson, Heidelberg, Germany). For intracellular staining, the cells were treated with FACS Permeabilizing Solution 2 (BD Biosciences, New Jersey, USA), pre-incubated with the Fc-receptor blocking agent and then incubated with anti-T2R38 as described above.

### Western blotting

Tumor cells (5×10^5^) were lysed with RIPA buffer (Tris-buffered saline containing 1 % Nonidet P-40, 0.5 % sodium deoxycholate, 0.1 % sodium dodecyl sulfate, 0.0004 % sodium azide, 0.2 M orthovanadate and 0.5 M phenylmethyl-sulfonyl fluoride). Of each sample 25 μl were mixed with 5 μl 5x Laemmli-buffer and applied to a SDS-Gel (9 %). Following blotting, the membrane was incubated in 5 % milk powder in TBS, containing 0.1 % Tween20. Of the antibody to T2R38 (sc-34294, Santa Cruz, Heidelberg, Germany) a 1:1000 dilution was used, of antigen peptide sc-34294P 0.5 μl/10 μl antibody. Secondary antibody was a POX conjugated mouse anti-goat IgG (Jackson Immuno Research, Suffolk, UK, 1:20000). Blots were developed using Amersham Prime Western Blotting detection Reagent (GE Healthcare, Freiburg, Germany).

### Analysis of signaling pathways

Cells were stimulated with either AHL-12 (Cayman Chemical Company, Michigan, USA) (10 to 100 μM) or PTU (100 μM to 10 mM, Sigma Aldrich, Schnelldorf, Germany) for the times indicated, then lysed with RIPA buffer and subjected to SDS-PAGE and Western blotting using the following antibodies: anti-pp38 (Biovision Inc, Milpitas, Ca, USA), anti-NFATc1, anti ERK1/2 (all obtained from Santa Cruz Biotechnology, Heidelberg, Germany), anti ABCB1 (Cell Signaling, Danvers, MA, USA). The membrane was incubated in 5 % milk powder in TBS, containing 0.1 % Tween20. Secondary antibody was a Pox conjugated goat anti rabbit IgG in a dilution of 1:15000 (Jackson Immuno Research, Pennsylvania, USA), respectively peroxidase-labelled anti-mouse IgG (Jackson Immuno Research).

### Laser scan microscopy

Cells were placed on cover slips, and fixed with 2 % PFA for 15 min. Then anti-T2R38 (6 μg) and the secondary antibody was added (Alexa Fluor 488 donkey anti-goat IgG (life technologies, Carlsbad, USA) were added, and for comparison goat IgG. In parallel, the antibody was added to the cells together with the peptide sc-34294P. The samples were mounted with Moviol (Sigma-Aldrich) and viewed by laser scan microscopy (Nikon) using a 40x objective.

For visualization of droplets, Nile red (Sigma-Aldrich) was added (10 μg /ml 20 min, room temperature). Alternatively, an antibody to perilipin-2 (Biozol, Eching, Germany).

### Isolation of lipid droplets

Essentially, the method described by Wan et al. [30] was used. In brief, cells were suspended in 3 ml ice cold disruption buffer (25 mM Tris-HCl pH 7.4, 5 mM EDTA, 1 mM EGTA, 0.2 mM PMSF, 50 μg /ml N- α -p-tosyl- L-lysin-chloromethyl-ketone (TLCK), 1 μg /ml leupeptin, 1 μg /ml pepstatin, 1μg /ml aprotinin) and lysed by nitrogen cavitation (10 min, 800 psi, 4°C). The lysate was mixed with an equal volume of 1.08 M sucrose in disruption buffer without TLCK, centrifuged for 30 min, 1000 g at 4°C to eliminate nuclei and intact cells and the applied to a sucrose density centrifugation. After ultracentrifugation (3.5 h, 34000 rpm, 4°C) the lipid droplets were in the fraction with 0.54 M sucrose. This fraction was diluted to 0.35 M sucrose and 0.15 M NaCl, overlaid again with 0.27 M, 0.135 M and 0 M sucrose for a second ultracentrifugation step (34000 rpm, 4°C, and 3.5 h). The lipid droplets were now in the fraction with 0.35 M sucrose. Droplets could be recognised by fluorescence microscopy after having incorporated Nile red (see above). For Western blotting the proteins were precipitated with methanol (sample : methanol 1 :4, 5 h,-20°C, followed by centrifugation for 30 min, 4°C, 16000g). The pellet was resuspended in Laemmli buffer (95°C for 10 min) and applied to a 12 % SDS-Gel. Western blotting was performed as described above.

### Patients and biopsies

Tissue of 88 resected pancreatic cancers (48 male patients, 40 female patients) was obtained after surgery and establishing the diagnosis of a pancreatic ductal adenocarcinoma. The mean age on day of surgery was 62.2 years (median: 65 years). All patients revealed a pT3 stage, 78 were diagnosed with lymph node metastasis (pN1), and 10 with distant organ metastasis (liver, inter-aorto-caval lymph nodes). The histological grade was G1: 4 patients, G2: 58 patients, and G3: 24 patients. Two patients received no histological grading, due to pre-operative neo-adjuvant therapy. 71 patients were diagnosed with an R1 stage (defined as tumor cells less than 1 mm from the peripancreatic margin). Follow up information was available of 72 patients (median survival in the T2R38 expression group: 623 days; in the non-expressing group 599 days). The study was approved by the local Ethics Committee of the University Hospital of Heidelberg. A written informed consent of all patients was obtained.

### Tissue staining

Tissue micorarrays with a core size of 1.5 mm in diameter were performed of patients with pancreatic ductal adenocarcinoma. Standard hematoxylin/eosin (H&E) staining was performed, followed by immunohistochemistry, using an anti human T2R38 (Santa Cruz, sc-34294, 1:100, and retrieval condition: pH 9.0). As secondary antibody the Dako EnVision kit (Dako) was used, followed by counter stain with hematoxylin.

Indirect immunofluorescence: Pancreatic cancer tissue (n=20 patients) was used. After deparaffinization and antigen retrieval (pH 9.0), the tissue sections were blocked with 1% bovine serum albumin in TBS, followed by incubation with the primary antibodies: goat anti human T2R38 (sc-34294, Santa Cruz, 1:100), and anti-perilipin-2 (Biozol, Eching, Germany). As secondary antibody the Alexa488 donkey anti goat (LifeTechnologies, Eugene, Oregon, USA 1:400) or Cy3 conjugated goat anti mouse IgG (Jackson Immuno Research, 1:300) were used, followed by nuclear staining with Hoechst dye.

### Statistical analysis

Statistical analysis was performed using GraphPad Prism software 4.0 (GraphPad Software Inc., LaJolla, Ca, USA) and the appropriate tests for the clinical correlations (Mann-Whitney-U) and Log-Rank-test (survival).

## References

[1] Siegel RL, Miller KD, Jemal A (2015). Cancer statistics, 2015. CA: a cancer journal for clinicians.

[2] Weinberg BA, Yabar CS, Brody JR, Pishvaian MJ (2015). Current Standards and Novel Treatment Options for Metastatic Pancreatic Adenocarcinoma. Oncology.

[3] Kleeff J, Beckhove P, Esposito I, Herzig S, Huber PE, Lohr JM, Friess H (2007). Pancreatic cancer microenvironment. International journal of cancer.

[4] Alam MS, Gaida MM, Bergmann F, Lasitschka F, Giese T, Giese NA, Hackert T, Hinz U, Hussain SP, Kozlov SV, Ashwell JD (2015). Selective inhibition of the p38 alternative activation pathway in infiltrating T cells inhibits pancreatic cancer progression. Nat Med.

[5] Shanahan F (2013). The colonic microbiota in health and disease. Current opinion in gastroenterology.

[6] Schwabe RF, Jobin C (2013). The microbiome and cancer. Nature reviews Cancer.

[7] Maurer S, Wabnitz GH, Kahle NA, Stegmaier S, Prior B, Giese T, Gaida MM, Samstag Y, Hansch GM (2015). Tasting Pseudomonas aeruginosa Biofilms: Human Neutrophils Express the Bitter Receptor T2R38 as Sensor for the Quorum Sensing Molecule N-(3-Oxododecanoyl)-l-Homoserine Lactone. Frontiers in immunology.

[8] Lee RJ, Xiong G, Kofonow JM, Chen B, Lysenko A, Jiang P, Abraham V, Doghramji L, Adappa ND, Palmer JN, Kennedy DW, Beauchamp GK, Doulias PT, Ischiropoulos H, Kreindler JL, Reed DR (2012). T2R38 taste receptor polymorphisms underlie susceptibility to upper respiratory infection. The Journal of clinical investigation.

[9] Tizzano M, Gulbransen BD, Vandenbeuch A, Clapp TR, Herman JP, Sibhatu HM, Churchill ME, Silver WL, Kinnamon SC, Finger TE (2010). Nasal chemosensory cells use bitter taste signaling to detect irritants and bacterial signals. Proceedings of the National Academy of Sciences of the United States of America.

[10] Shah AS, Ben-Shahar Y, Moninger TO, Kline JN, Welsh MJ (2009). Motile cilia of human airway epithelia are chemosensory. Science.

[11] Viswanathan VK (2013). Sensing bacteria, without bitterness?. Gut microbes.

[12] Adler E, Hoon MA, Mueller KL, Chandrashekar J, Ryba NJ, Zuker CS (2000). A novel family of mammalian taste receptors. Cell.

[13] Beller M, Thiel K, Thul PJ, Jackle H (2010). Lipid droplets: a dynamic organelle moves into focus. FEBS letters.

[14] Meyerhof W, Behrens M, Brockhoff A, Bufe B, Kuhn C (2005). Human bitter taste perception. Chemical senses.

[15] Ji M, Su X, Su X, Chen Y, Huang W, Zhang J, Gao Z, Li C, Lu X (2014). Identification of Novel Compounds for Human Bitter Taste Receptors. Chemical biology & drug design.

[16] Singh N, Chakraborty R, Bhullar RP, Chelikani P (2014). Differential expression of bitter taste receptors in non-cancerous breast epithelial and breast cancer cells. Biochemical and biophysical research communications.

[17] Rozengurt N, Wu SV, Chen MC, Huang C, Sternini C, Rozengurt E (2006). Colocalization of the alpha-subunit of gustducin with PYY and GLP-1 in L cells of human colon. American journal of physiology Gastrointestinal and liver physiology.

[18] Wu SV, Rozengurt N, Yang M, Young SH, Sinnett-Smith J, Rozengurt E (2002). Expression of bitter taste receptors of the T2R family in the gastrointestinal tract and enteroendocrine STC-1 cells. Proceedings of the National Academy of Sciences of the United States of America.

[19] Behrens M, Born S, Redel U, Voigt N, Schuh V, Raguse JD, Meyerhof W (2012). Immunohistochemical detection of TAS2R38 protein in human taste cells. PloS one.

[20] Straub BK, Gyoengyoesi B, Koenig M, Hashani M, Pawella LM, Herpel E, Mueller W, Macher-Goeppinger S, Heid H, Schirmacher P (2013). Adipophilin/perilipin-2 as a lipid droplet-specific marker for metabolically active cells and diseases associated with metabolic dysregulation. Histopathology.

[21] Kahle NA, Brenner-Weiss G, Overhage J, Obst U, Hansch GM (2013). Bacterial quorum sensing molecule induces chemotaxis of human neutrophils *via* induction of p38 and leukocyte specific protein 1 (LSP1). Immunobiology.

[22] Kravchenko VV, Kaufmann GF, Mathison JC, Scott DA, Katz AZ, Wood MR, Brogan AP, Lehmann M, Mee JM, Iwata K, Pan Q, Fearns C, Knaus UG, Meijler MM, Janda KD, Ulevitch RJ (2006). N-(3-oxo-acyl)homoserine lactones signal cell activation through a mechanism distinct from the canonical pathogen-associated molecular pattern recognition receptor pathways. The Journal of biological chemistry.

[23] Singh N, Vrontakis M, Parkinson F, Chelikani P (2011). Functional bitter taste receptors are expressed in brain cells. Biochemical and biophysical research communications.

[24] Jeon TI, Seo YK, Osborne TF (2011). Gut bitter taste receptor signalling induces ABCB1 through a mechanism involving CCK. The Biochemical journal.

[25] Rozengurt E (2006). Taste receptors in the gastrointestinal tract. I. Bitter taste receptors and alpha-gustducin in the mammalian gut. American journal of physiology Gastrointestinal and liver physiology.

[26] Farese RV, Walther TC (2009). Lipid droplets finally get a little R-E-S-P-E-C-T. Cell.

[27] Martin S, Parton RG (2006). Lipid droplets: a unified view of a dynamic organelle. Nature reviews Molecular cell biology.

[28] Welte MA (2007). Proteins under new management: lipid droplets deliver. Trends in cell biology.

[29] Straub BK, Herpel E, Singer S, Zimbelmann R, Breuhahn K, Macher-Goeppinger S, Warth A, Lehmann-Koch J, Longerich T, Heid H, Schirmacher P (2010). Lipid droplet-associated PAT-proteins show frequent and differential expression in neoplastic steatogenesis. Modern pathology.

[30] Wan HC, Melo RC, Jin Z, Dvorak AM, Weller PF (2007). Roles and origins of leukocyte lipid bodies: proteomic and ultrastructural studies. FASEB journal.

[31] Karlsson T, Musse F, Magnusson KE, Vikstrom E (2012). N-Acylhomoserine lactones are potent neutrophil chemoattractants that act *via* calcium mobilization and actin remodeling. Journal of leukocyte biology.

[32] Vikstrom E, Magnusson KE, Pivoriunas A (2005). The Pseudomonas aeruginosa quorum-sensing molecule N-(3-oxododecanoyl)-L-homoserine lactone stimulates phagocytic activity in human macrophages through the p38 MAPK pathway. Microbes and infection.

[33] Zimmermann S, Wagner C, Muller W, Brenner-Weiss G, Hug F, Prior B, Obst U, Hansch GM (2006). Induction of neutrophil chemotaxis by the quorum-sensing molecule N-(3-oxododecanoyl)-L-homoserine lactone. Infection and immunity.

[34] Wagner C, Zimmermann S, Brenner-Weiss G, Hug F, Prior B, Obst U, Hansch GM (2007). The quorum-sensing molecule N-3-oxododecanoyl homoserine lactone (3OC12-HSL) enhances the host defence by activating human polymorphonuclear neutrophils (PMN). Analytical and bioanalytical chemistry.

[35] Hansch GM (2012). Editorial: molecular eavesdropping: phagocytic cells spy on bacterial communication. Journal of leukocyte biology.

[36] Lee RJ, Cohen NA (2015). Taste receptors in innate immunity. Cellular and molecular life sciences.

[37] Lee RJ, Cohen NA (2015). Role of the bitter taste receptor T2R38 in upper respiratory infection and chronic rhinosinusitis. Current opinion in allergy and clinical immunology.

[38] Clark AA, Liggett SB, Munger SD (2012). Extraoral bitter taste receptors as mediators of off-target drug effects. FASEB journal.

[39] Ambudkar SV, Kim I-W, Sauna ZE (2006). The power of the pump: Mechanisms of action of P-glycoprotein (ABCB1). European Journal of Pharmaceutical Sciences.

[40] Ambudkar SV, Sauna ZE, Gottesman MM, Szakacs G (2005). A novel way to spread drug resistance in tumor cells: functional intercellular transfer of P-glycoprotein (ABCB1). Trends in Pharmacological Sciences.

[41] Carrai M, Steinke V, Vodicka P, Pardini B, Rahner N, Holinski-Feder E, Morak M, Schackert HK, Gorgens H, Stemmler S, Betz B, Kloor M, Engel C, Buttner R, Naccarati A, Vodickova L (2011). Association between TAS2R38 gene polymorphisms and colorectal cancer risk: a case-control study in two independent populations of Caucasian origin. PloS one.

[42] Bravi F, Bosetti C, Tavani A, Gallus S, La Vecchia C (2013). Coffee reduces risk for hepatocellular carcinoma: an updated meta-analysis. Clinical gastroenterology and hepatology.

[43] Gao F, Li M, Liu WB, Zhou ZS, Zhang R, Li JL, Zhou KC (2015). Epigallocatechin gallate inhibits human tongue carcinoma cells *via* HK2mediated glycolysis. Oncology reports.

[44] Lamy V, Roussi S, Chaabi M, Gosse F, Schall N, Lobstein A, Raul F (2007). Chemopreventive effects of lupulone, a hop {beta}-acid, on human colon cancer-derived metastatic SW620 cells and in a rat model of colon carcinogenesis. Carcinogenesis.

[45] Singh N, Chakraborty R, Bhullar RP, Chelikani P (2014). Differential expression of bitter taste receptors in non-cancerous breast epithelial and breast cancer cells. Biochemical and biophysical research communications.

